# MSAFusion: A Lightweight Multispectral Pedestrian Detection Network with Multi-Scale and Adaptive Feature Fusion

**DOI:** 10.3390/jimaging12060246

**Published:** 2026-05-30

**Authors:** Yang Song, Xin Zuo, Chenyu Qu, Qiang Qian, Dengbiao Jiang

**Affiliations:** School of Computer Science, Jiangsu University of Science and Technology, Zhenjiang 212003, China; 231710701114@stu.just.edu.cn (Y.S.); chenyuqu92@gmail.com (C.Q.); qianqiang_just@just.edu.cn (Q.Q.); jiangdengb@just.edu.cn (D.J.)

**Keywords:** RGB–thermal pedestrian detection, multispectral pedestrian detection, small-object detection, multi-scale feature refinement, adaptive feature recalibration, lightweight architecture

## Abstract

Robust multispectral pedestrian detection remains challenging in complex environments such as those with low illumination, strong thermal contrast, and background clutter. Although RGB–thermal fusion provides complementary cues, lightweight detectors often suffer from unstable feature representation across scales and insufficient control over modality-biased responses during fusion, which can degrade localization accuracy and weaken the detection of small or distant pedestrians. To address these issues, we develop a lightweight stage-wise RGB–thermal fusion pipeline that integrates pre-fusion feature refinement, cross-modal interaction, and post-fusion adaptive recalibration. Specifically, a Multi-scale Feature Refinement (MSFR) module is proposed at the mid-level to enhance modality-specific representations by jointly modeling local details and contextual information, thereby reducing scale-sensitive noise before interaction. An established Cross-Modality Fusion Transformer (CFT) is then adopted to promote semantic correspondence between RGB and thermal features. After interaction, an Adaptive Feature Recalibration (AFR) module is introduced to suppress background-dominated and modality-biased responses through lightweight channel-wise adjustment. Extensive experiments on three public RGB–thermal benchmarks, including the pedestrian-focused KAIST and LLVIP datasets together with the FLIR-aligned road-scene benchmark, demonstrate that the proposed method achieves a favorable accuracy–efficiency trade-off, delivering consistent improvements over the lightweight baseline while maintaining a compact architecture and real-time inference capability.

## 1. Introduction

Multispectral imaging [1] provides a richer and more reliable description of real-world scenes by integrating complementary sensing mechanisms, effectively compensating for the limitations of visible imagery [2] under low illumination, severe clutter, and strong interference. In computer vision [3], multispectral perception has become increasingly important [4], benefiting a wide range of downstream tasks such as object detection [5], semantic segmentation [6], and visual tracking [7]. Among these tasks, pedestrian detection is particularly critical for intelligent transportation, autonomous driving, and public safety systems. Although object detection has achieved remarkable progress in recent years [8], detectors relying solely on RGB images still suffer from degraded performance in adverse environments such as nighttime scenes, fog, and abrupt illumination changes [9]. In such cases, appearance cues become unreliable, and background noise may dominate feature responses, leading to missed detections and unstable localization. By contrast, RGB–thermal multispectral detection can exploit complementary information from the two modalities: RGB images provide rich color, texture, and edge details under normal illumination [10], while thermal images preserve relatively stable target silhouettes when visible cues are severely degraded [11]. This complementarity makes multispectral pedestrian detection a promising solution for robust perception in challenging conditions [12].

Despite its advantages, reliable RGB–thermal pedestrian detection remains nontrivial. The key challenge lies not only in whether visible and thermal features are fused, but also in how and where fusion is performed to preserve feature stability, cross-modal complementarity, and computational efficiency across diverse scenes [13]. Existing multispectral detectors [14] mainly follow convolution-based fusion, attention-enhanced fusion, or Transformer-based interaction paradigms. Mid-level fusion has been shown to be more effective than purely early or late fusion in many cases, yet convolutional operations primarily emphasize local interactions and are often insufficient for modeling long-range cross-modal dependencies. More recent methods have introduced spatial or channel attention to strengthen salient modality cues [15], but many lightweight designs still rely on direct one-shot fusion, fixed enhancement heuristics, or relatively limited response recalibration. As a result, noisy modality-specific features may be propagated into the fusion stage, and indiscriminate reweighting may further amplify background-dominated or modality-biased channels. These issues are especially evident in lightweight detectors, where restricted representational capacity makes the model more sensitive to scale variation, sensor noise, and cluttered backgrounds. Consequently, achieving a favorable balance among feature stability, cross-modal interaction quality, and inference efficiency remains a central challenge, particularly for small or distant pedestrians [16].

From this perspective, existing studies have devoted considerable effort to designing stronger fusion operators, but comparatively less attention has been paid to the coordination of three closely related stages in lightweight multispectral detection: improving modality-specific feature quality before interaction, establishing effective cross-modal correspondence during interaction, and recalibrating fused responses after interaction. If the features entering the interaction module are unstable, even a powerful fusion mechanism may inherit scale-sensitive noise or modality imbalance. Likewise, without proper post-fusion recalibration, background-dominated activations and redundant responses may persist and interfere with subsequent multi-scale aggregation. Therefore, a lightweight detector can benefit not only from cross-modal interaction itself but also from a more structured fusion process surrounding that interaction.

To address these issues, we develop a lightweight stage-wise RGB–thermal fusion pipeline for pedestrian detection. Rather than proposing a new Transformer-based fusion operator, this work investigates how an established cross-modal interaction mechanism can be more effectively embedded into a compact detector through coordinated pre-fusion refinement and post-fusion recalibration. Specifically, modality-specific features are first refined at a mid-level stage to alleviate scale inconsistency and suppress noisy responses before interaction. Cross-modal semantic correspondence is then modeled at a higher-level stage using an adopted Transformer-based interaction mechanism. After interaction, a lightweight channel-recalibration module is introduced to rebalance fused responses and suppress redundant or background-dominated activations before multi-scale aggregation. Under this design, two dedicated components are proposed: a Multi-scale Feature Refinement (MSFR) module, which strengthens mid-level RGB and thermal representations with limited overhead, and an Adaptive Feature Recalibration (AFR) module, which performs efficient post-fusion channel redistribution using complementary statistical cues. In this way, the proposed method focuses on optimizing the fusion pipeline itself rather than relying on a heavier backbone or a more complex interaction operator, thereby improving the accuracy–efficiency trade-off of lightweight multispectral pedestrian detection.

The main contributions of this work can be summarized as follows:

(1) We establish a lightweight stage-wise RGB–thermal fusion pipeline for pedestrian detection that explicitly organizes multispectral integration into pre-fusion feature refinement, cross-modal interaction, and post-fusion recalibration to improve feature stability and modality balance in compact detectors.
(2) We propose two lightweight plug-in modules, namely Multi-scale Feature Refinement (MSFR) and Adaptive Feature Recalibration (AFR). MSFR enhances modality-specific features before interaction by jointly modeling local details and contextual information, while AFR recalibrates fused responses after interaction to suppress background-dominated and modality-biased activations.
(3) Extensive experiments on three public RGB–thermal benchmarks, including the pedestrian-focused KAIST [17] and LLVIP [18] datasets together with the FLIR-aligned road-scene benchmark [19], demonstrate that the proposed method consistently improves the lightweight baseline and achieves a favorable balance among detection accuracy, model compactness, and real-time inference capability.

## 2. Related Work

### 2.1. Multispectral Object Detection Based on Deep Learning

Multispectral object detection aims to improve perception robustness in challenging environments, such as nighttime scenes, occlusion, and background clutter, by jointly exploiting visible (RGB) and thermal infrared (T) modalities. With the development of deep learning, this field has gradually evolved from early shallow fusion strategies to more advanced architectures involving multi-branch representation learning, attention-guided fusion, and Transformer-based cross-modal interaction. Nevertheless, due to the substantial differences in imaging mechanisms, feature distributions, and response reliability between RGB and thermal data, achieving stable, complementary, and efficient multispectral fusion remains a fundamental challenge.

Early studies mainly focused on determining an appropriate fusion stage. Liu et al. [20] proposed the representative Halfway Fusion architecture, which integrates visible and thermal features at an intermediate layer, and demonstrated the advantage of mid-level fusion over purely early or late fusion. However, such static fusion schemes treat modality contributions in a relatively fixed manner and are therefore less capable of adapting to scene-dependent illumination variation or modality reliability changes. To improve fusion flexibility, Zhang et al. [21] introduced a guided attention-based fusion strategy to emphasize salient regions across modalities. Although these approaches enhance local feature representation, they still rely predominantly on convolutional modeling and remain limited in capturing long-range semantic correspondence between RGB and thermal features.

To address this limitation, Transformer-based interaction mechanisms were recently introduced into multispectral detection. Fang et al. [22] proposed the Cross-Modality Fusion Transformer (CFT), which establishes semantic correspondence between RGB and thermal features through attention-based cross-modal interaction. Subsequent methods, such as MCAFNet [23], further enhance multimodal representation by incorporating richer contextual encoding and multi-scale interaction. These approaches improve the ability to model global dependencies and modality complementarity. However, many of them focus primarily on the interaction operator itself, while paying relatively less attention to the quality of modality-specific features before interaction and the suppression of redundant or biased responses after interaction. As a result, unstable features may still be propagated into the fusion stage, and background-dominated responses may persist in cluttered or low-resolution scenes.

Another important research direction concerns selective feature enhancement and efficient multispectral deployment. Fang and Wang [24] proposed Cross-Modality Attentive Feature Fusion (CMAFF), which treats modality-shared and modality-specific information differently through common-modality and differential-modality attention branches. This design highlights the importance of selective interaction rather than indiscriminate feature fusion. Cui et al. [25] further introduced a Selective cross-modal Interaction and Aggregation (SIA) framework for multispectral remote sensing object detection, aiming to reduce irrelevant information introduced during long-range dependency modeling and heterogeneous feature aggregation. These studies demonstrate that cross-modal fusion benefits from selective information exchange and modality-aware feature organization. However, they are mainly developed for remote sensing scenarios or general multispectral object detection, and they do not explicitly investigate a lightweight pedestrian detection pipeline that coordinates pre-fusion feature refinement, cross-modal interaction, and post-fusion recalibration in a stage-wise manner.

Lightweight multispectral detection has also attracted growing attention. Jin et al. [26] proposed FCMNet, which enhances edge-related information through frequency-domain cross-modal attention. Zhang et al. [27] integrated Coordinate Attention and CARAFE-based feature reassembly into a YOLOv5 framework to improve spatial precision while controlling complexity, whereas Li et al. [28] employed Shuffle Attention, GSConv, and depthwise decoupled heads to improve inference efficiency. These methods indicate that lightweight multispectral detectors can benefit from efficient attention or feature enhancement modules. However, their limited representational capacity makes them more vulnerable to scale variation, noisy modality-specific features, and modality imbalance. In addition, excessive branch construction or repeated fusion operations may introduce redundancy and parameter growth, as observed in recent modular fusion designs such as MambaDFuse [29]. Therefore, improving fusion effectiveness without substantially increasing model complexity remains an important issue for lightweight RGB–thermal pedestrian detection.

Overall, existing multispectral detection methods have made significant progress in fusion stage design, cross-modal interaction, and efficient deployment. Nevertheless, three limitations remain particularly relevant to lightweight pedestrian detection: (i) modality-specific features are often directly fused without sufficient stabilization beforehand; (ii) cross-modal interaction is usually treated as the dominant design focus, while the coordination between pre-interaction and post-interaction processing is less explored; and (iii) fused features may still contain background-dominated or modality-biased responses that affect subsequent multi-scale aggregation. These observations motivate the stage-wise fusion design adopted in this work.

### 2.2. Attention and Feature Recalibration Mechanisms

Attention mechanisms have been widely used in object detection and multimodal feature learning to selectively emphasize informative channels or spatial regions. The Squeeze-and-Excitation (SE) module [30] models global inter-channel dependencies through channel-wise recalibration, while CBAM [31] sequentially applies channel and spatial attention to strengthen discriminative responses. For lightweight attention design, ECA-Net [32] captures local cross-channel interaction without dimensionality reduction, Coordinate Attention [33] embeds positional information into channel attention, and SimAM [34] performs parameter-free saliency enhancement based on an energy formulation. These modules have demonstrated strong effectiveness in conventional vision tasks by improving feature selectivity and suppressing irrelevant responses.

However, directly transferring generic attention mechanisms to multispectral detection is not always sufficient. In RGB–thermal detection, modality reliability changes dynamically with illumination, background complexity, and sensor characteristics. After cross-modal interaction, fused features may exhibit channel responses dominated by one modality, over-activated background regions, or weak responses for small pedestrians. Conventional attention mechanisms are generally designed for single-stream or generic feature enhancement and are not specifically tailored to recalibrate post-fusion multispectral representations. Therefore, although they can improve feature discriminability, they may be less effective in addressing the modality-biased and background-dominated responses that emerge after RGB–thermal interaction.

Motivated by this issue, the proposed Adaptive Feature Recalibration (AFR) module is designed as a lightweight post-fusion recalibration mechanism rather than a generic attention block. AFR jointly exploits complementary global statistics and local channel interaction to adaptively redistribute the importance of fused responses. Its objective is to suppress redundant or background-dominated activations while preserving pedestrian-relevant channels after cross-modal interaction. In this sense, AFR differs from conventional attention modules by focusing specifically on the stabilization of fused multispectral features before multi-scale aggregation.

### 2.3. Distinction from Closely Related Fusion Strategies

Several recent studies are conceptually related to the present work because they also emphasize selective interaction, progressive processing, or modality-aware fusion. Nevertheless, their technical objectives, architectural roles, and target tasks differ from ours.

CFT [22] introduces Transformer-based RGB–thermal interaction and serves as the adopted cross-modal correspondence mechanism in our framework. Our contribution does not lie in redesigning CFT itself, but in investigating how such an established interaction block can be more effectively embedded into a lightweight detector through coordinated pre-fusion refinement and post-fusion recalibration. CMAFF [24] and SIA [25] both highlight the importance of selective feature interaction and modality-aware aggregation. Specifically, CMAFF focuses on separating common and differential modality cues, while SIA targets selective cross-modal interaction and aggregation for multispectral remote sensing object detection. In contrast, our method is specifically designed for lightweight RGB–thermal pedestrian detection and organizes the detection pipeline into three sequential stages: modality-specific refinement, adopted Transformer-based interaction, and post-fusion adaptive recalibration.

In addition, Hu et al. [35] proposed a Cross-Modal Fusion and Progressive Decoding Network (CPNet) for RGB-D salient object detection. Although CPNet also adopts a progressive processing philosophy, it addresses a different task and modality setting. Its main focus lies in RGB-D saliency prediction and decoder-side progressive reconstruction, rather than lightweight RGB–thermal pedestrian detection. Therefore, their similarity is mainly conceptual at the level of progressive organization, whereas their technical objective, network role, and detection problem are substantially different from those of our work.

More specifically, the distinction between our method and the above studies lies in both the function of each component and the overall optimization objective. CFT primarily focuses on designing a Transformer-based interaction operator for RGB–thermal correspondence modeling, whereas our work adopts CFT as an intermediate interaction block and instead concentrates on improving the feature conditions before and after interaction. CMAFF and SIA emphasize selective cross-modal information exchange or aggregation, but they do not explicitly construct a lightweight pedestrian detection pipeline in which modality-specific features are refined before interaction and fused responses are recalibrated afterward. CPNet follows a progressive processing philosophy, yet it is developed for RGB-D salient object detection and mainly performs progressive decoder-side reconstruction rather than stage-wise enhancement for RGB–thermal pedestrian detection. Therefore, our method is distinguished by a lightweight fusion organization that coordinates pre-fusion refinement, adopted cross-modal interaction, and post-fusion recalibration within a unified pedestrian detection framework.

Taken together, these studies confirm the importance of selective cross-modal modeling and staged feature processing. Building on these insights, our work focuses on improving the accuracy–efficiency trade-off of lightweight RGB–thermal pedestrian detection through a structured refinement–interaction–recalibration pipeline, in which the proposed MSFR and AFR modules serve as the main architectural contributions.

## 3. Method

### 3.1. Architecture

**Figure 1:**
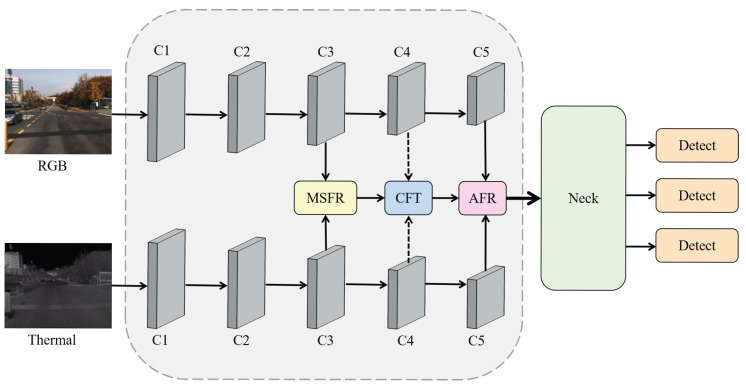
The network adopts a dual-branch structure that extracts features from both RGB and thermal images. C1–C5 denote feature maps from different stages. MSFR and AFR represent the proposed Multi-Scale Feature Refinement and Adaptive Feature Recalibration modules, respectively, while CFT denotes the adopted Cross-Modality Fusion Transformer. These modules are embedded into different feature levels to realize a stage-wise refinement–interaction–recalibration process. The resulting features are aggregated by the neck across multiple scales and finally processed by the head to generate the detection results.

This work targets RGB–thermal multispectral pedestrian detection and aims to address the instability of cross-modal fusion under complex illumination conditions and scale variations. As illustrated in Figure 1, we design a lightweight detection framework composed of dual-stream backbones, stage-wise cross-modal enhancement modules, and a multi-scale detection head. The overall architecture follows a structured fusion principle that first stabilizes single-modality representations, then establishes cross-modal correspondence, and finally adaptively recalibrates fused features, supporting efficient multispectral feature modeling with limited additional overhead.

Given paired RGB and thermal inputs, the two modalities are first processed by dedicated backbones to extract multi-scale feature representations at stages l∈{1,2,3,4,5}. Let $I_{R}\in R^{H\times W\times 3}$ and $I_{T}\in R^{H\times W\times 1}$ denote the RGB and thermal inputs, respectively. When the thermal stream is replicated to three channels, $I_{T}\in R^{H\times W\times 3}$ is used. The feature extraction process of the dual backbones is defined in Equation (1).

(1) $$\begin{array}{cc} F_{R}^{l} & =\Psi_{R}^{l}\left(I_{R}\right)\\ F_{T}^{l} & =\Psi_{T}^{l}\left(I_{T}\right) \end{array}$$

where $\Psi_{R}^{l}$ and $\Psi_{T}^{l}$ denote the *l*-th stage of the RGB and thermal backbones, producing feature maps $F_{R}^{l},F_{T}^{l}\in R^{H_{l}\times W_{l}\times C_{l}}$.

Before cross-modal interaction, the RGB and thermal features at stage C3 are independently refined by the Multi-scale Feature Refinement (MSFR) module to improve single-modality representation quality and reduce scale-sensitive noise. The refined features are then concatenated along the channel dimension to form a unified C3 representation, as defined in Equation (2).

(2) $${\tilde{F}}^{3}=\mathrm{Concat}\;\left({\tilde{F}}_{R}^{3},\;{\tilde{F}}_{T}^{3}\right)$$

where ${\tilde{F}}_{R}^{3}$ and ${\tilde{F}}_{T}^{3}$ denote the refined RGB and thermal features at stage C3, respectively, and Concat(·) denotes channel-wise concatenation.

At stage C4, cross-modal interaction is modeled by an adopted Cross-Modality Fusion Transformer (CFT) [22], which establishes semantic correspondence between the RGB and thermal representations, as formulated in Equation (3).

(3) $$F_{\times }^{4}=\mathrm{CFT}\;\left(F_{R}^{4},\;F_{T}^{4}\right)$$

where $F_{R}^{4}$ and $F_{T}^{4}$ denote the RGB and thermal features at stage C4, respectively, and $F_{\times }^{4}$ is the fused output generated by CFT.

Although Transformer-based interaction facilitates cross-modal correspondence modeling, the fused features may still retain redundant responses or modality-biased activations under illumination variation and background clutter. To address this issue, an Adaptive Feature Recalibration (AFR) module is introduced at stage C5 to perform post-fusion channel recalibration on the fused representation. At the architectural level, this process is expressed as

(4) $${\hat{F}}^{5}=\mathrm{AFR}\;\left(F_{\times }^{5}\right)$$

where $F_{\times }^{5}$ denotes the fused feature representation at stage C5, AFR(·) represents the post-fusion recalibration operation detailed in Section 3.4, and ${\hat{F}}^{5}$ is the recalibrated output forwarded to the neck.

Finally, the multi-level features obtained from different stages are aggregated by the neck network to form a unified representation for detection. Specifically, the refined C3 feature, the fused C4 feature, and the recalibrated C5 feature are jointly processed by the neck and detection head, as formulated in Equation (5).

(5) $$Y=\varphi_{\mathrm{head}}\;\left(\varphi_{\mathrm{neck}}\left(\{{\tilde{F}}^{3},\;F_{\times }^{4},\;{\hat{F}}^{5}\}\right)\right)$$

where $\varphi_{\mathrm{neck}}$ and $\varphi_{\mathrm{head}}$ denote the neck network and detection head, respectively, which jointly process the aggregated features $\left\{{\tilde{F}}^{3},F_{\times }^{4},{\hat{F}}^{5}\right\}$ to produce the final detection output Y.

This stage-wise organization is intended to support the representation of small and distant pedestrians while preserving a lightweight design and compatibility with the subsequent detection pipeline.

### 3.2. Multi-Scale Feature Refinement (MSFR)

In challenging scenarios such as nighttime environments, backlighting, and weak-texture backgrounds, pedestrians often appear at small scales with low contrast and fragmented boundaries. Under these conditions, directly introducing cross-modal interaction may allow noisy or scale-sensitive modality-specific responses to propagate into later fusion stages. To alleviate this issue, we introduce a Multi-scale Feature Refinement (MSFR) module at stage C3, where RGB and thermal features are refined independently before entering the cross-modal interaction stage. The C3 level is selected because it retains relatively fine spatial information that is useful for small and distant pedestrians, while providing richer semantic abstraction than shallower backbone stages.

**Figure 2:**
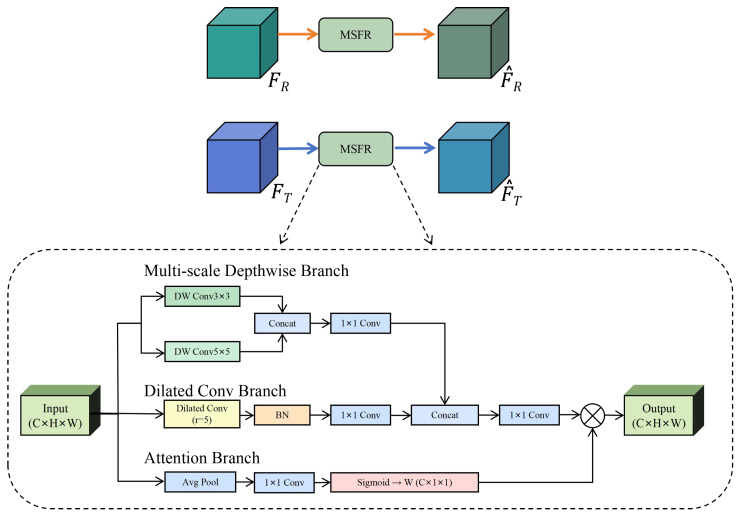
Illustration of the proposed MSFR module. In the upper row, RGB and thermal features at stage C3 are independently refined by MSFR to obtain enhanced modality-specific representations before subsequent cross-modal interaction. The lower row presents the internal structure of MSFR. Specifically, the multi-scale depthwise branch extracts local and mid-scale texture information, the dilated convolution branch enlarges the receptive field to encode broader contextual cues, and the attention branch produces channel-wise weights for adaptive feature modulation. The final output is obtained by aggregating these branches and applying channel recalibration, yielding a refined representation for downstream fusion.

MSFR is designed to enhance mid-level representations by jointly modeling local texture details, mid-scale structural cues, and broader contextual information with limited computational overhead. As illustrated in Figure 2, the module consists of three parallel components: a multi-scale depthwise branch for lightweight local and mid-scale feature extraction, a dilated-context branch for expanding the receptive field without reducing spatial resolution, and a channel-attention modulation branch for adaptive feature reweighting. All branches operate at the same spatial resolution and stride, which facilitates subsequent aggregation and maintains compatibility with the following backbone stage. For visual simplicity, the refined RGB and thermal outputs in Figure 2 are schematically denoted as ${\hat{F}}_{R}$ and ${\hat{F}}_{T}$, corresponding to $Y_{\mathrm{MSFR}}^{R}$ and $Y_{\mathrm{MSFR}}^{T}$, as defined below.

Let the input feature be $X\in R^{H\times W\times C}$. The multi-scale depthwise branch employs depthwise-separable convolutions with different kernel sizes to capture complementary spatial responses. Specifically, the 3×3 branch is intended to preserve fine local structures such as pedestrian contours and edges, whereas the 5×5 branch provides a moderately enlarged receptive field for mid-scale contextual patterns, as defined in Equations (6) and (7).

(6) $$\begin{array}{cc} F_{3} & =f_{\mathrm{dw}}^{3\times 3}\left(X;\;W_{\mathrm{dw}3}\right) \end{array}$$

(7) $$\begin{array}{cc} F_{5} & =f_{\mathrm{dw}}^{5\times 5}\left(X;\;W_{\mathrm{dw}5}\right) \end{array}$$

where $f_{\mathrm{dw}}^{k\times k}\left(\cdot ;\cdot \right)$ denotes a depthwise-separable convolution with kernel size k×k, and $F_{3}$ and $F_{5}$ represent fine-scale and mid-scale feature responses, respectively.

The two responses are concatenated along the channel dimension and projected using a 1×1 convolution to synthesize a unified multi-scale texture representation, as formulated in Equation (8).

(8) $$\begin{array}{c} F_{\mathrm{ms}}=\phi_{1\times 1}\left(\left[F_{3}\;∥\;F_{5}\right];\;W_{\mathrm{ms}}\right) \end{array}$$

where [·∥·] denotes channel-wise concatenation, and $\phi_{1\times 1}$ represents a 1×1 projection convolution.

Complementary to the texture-oriented branch, the dilated-context branch is introduced to enlarge the receptive field and provide additional contextual support without reducing spatial resolution. Its output is computed using a 3×3 dilated convolution with dilation rate *d*, followed by batch normalization, as formulated in Equation (9).

(9) $$\begin{array}{c} F_{D}=\mathrm{BN}\;\left(f_{k=3,\;d}^{\mathrm{dilated}}\left(X;\;W_{d}\right)\right) \end{array}$$

where we set d=5 in all experiments. Under this setting, the effective receptive field expands to approximately 11×11, allowing the module to incorporate wider contextual cues that may be useful under sparse texture, partial occlusion, or thermal response ambiguity.

Since dilated sampling may introduce discontinuous spatial responses, the dense local cues provided by the depthwise branch serve as a complementary reference at the same resolution. In addition, the subsequent 1×1 channel mixing helps integrate the outputs of different branches before recalibration. Symmetric padding is used to preserve spatial alignment, which is important for the cross-modal interaction performed at the subsequent C4 stage.

To adaptively regulate the contribution of different channels, MSFR further incorporates a lightweight channel-attention modulation branch. After the multi-scale and contextual responses are prepared, global statistics of the original input feature *X* are used to generate channel-wise modulation weights. Specifically, global average pooling compresses *X* into a channel descriptor $z\in R^{C}$, which is then transformed by two linear layers with a nonlinear activation to produce the channel weight vector $s\in R^{C}$, as expressed in Equation (10).

(10) $$\begin{array}{c} s=\sigma \;\left(W_{2}\;\delta \left(W_{1}z\right)\right) \end{array}$$

where $W_{1}$ and $W_{2}$ are the learnable weights of the two linear layers, δ denotes the ReLU activation, and σ is the sigmoid function. The resulting vector *s* is reshaped to C×1×1 for channel-wise broadcasting. Using the original backbone feature *X* to estimate the gating weights provides a stable global descriptor for feature modulation and avoids relying solely on sparsely sampled contextual responses.

Finally, the multi-scale texture feature $F_{\mathrm{ms}}$ and the dilated-context feature $F_{D}$ are concatenated, projected by a 1×1 convolution, modulated by the channel weights, and normalized to generate the refined output, as summarized in Equation (11). The projection restores the aggregated representation to the channel dimension required by the modulation weights and the subsequent backbone stage.

(11) $$\begin{array}{c} Y_{\mathrm{MSFR}}=\mathrm{BN}\;\left(s⊙\phi_{1\times 1}\left(\left[F_{\mathrm{ms}}\;∥\;F_{D}\right];\;W_{\mathrm{out}}\right)\right) \end{array}$$

where ⊙ denotes element-wise multiplication, $W_{\mathrm{out}}$ is the weight of the output projection convolution, and BN denotes batch normalization.

The resulting output $Y_{\mathrm{MSFR}}\in R^{H\times W\times C}$ preserves spatial resolution and channel dimensionality, thereby maintaining compatibility with the subsequent stage. When applied to the RGB and thermal branches, the refined features are denoted $Y_{\mathrm{MSFR}}^{R}$ and $Y_{\mathrm{MSFR}}^{T}$, respectively. These representations are then forwarded to the next backbone stage to produce $F_{R}^{4}$ and $F_{T}^{4}$, upon which the adopted CFT performs cross-modal interaction. In this way, MSFR serves as a pre-interaction refinement module that aims to provide better-conditioned modality-specific features for the subsequent fusion pipeline, and its contribution is further examined in the experimental section.

### 3.3. Adopted Cross-Modality Fusion Transformer (CFT)

At stage C4, we adopt the Cross-Modality Fusion Transformer (CFT) as an intermediate interaction component to establish semantic correspondence between the RGB and thermal streams. CFT is not redesigned in this work; its internal architecture and computation flow are preserved following the original formulation. Only the input–output interfaces and feature dimensions are adjusted where necessary to ensure compatibility with the MSFR-refined features from C3 and the subsequent Adaptive Feature Recalibration (AFR) stage at C5. This setting allows us to investigate how an established cross-modal interaction mechanism can be more effectively embedded into a lightweight refinement–interaction–recalibration pipeline, while attributing the proposed architectural contributions to the surrounding MSFR and AFR modules rather than to modifications to the Transformer itself.

**Figure 3:**
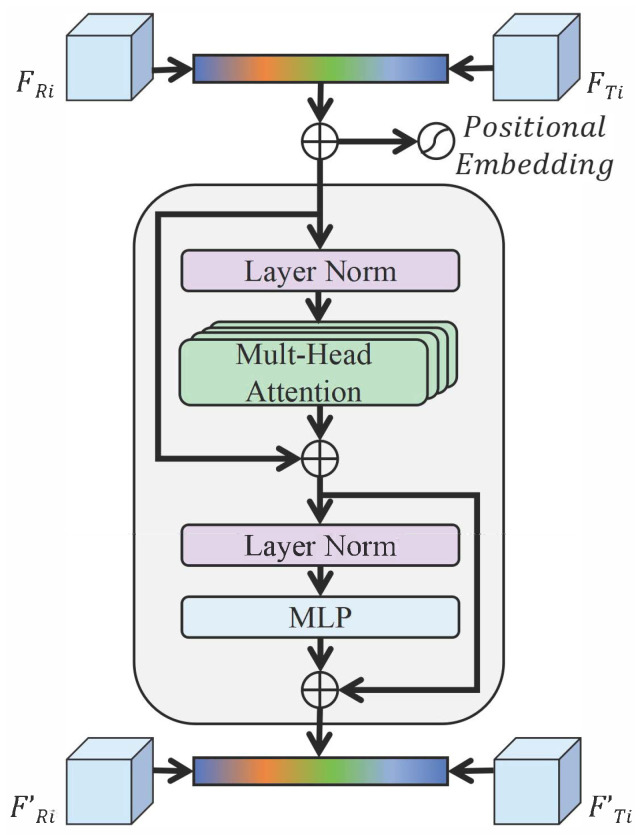
CFT block (adopted). Two branches (RGB/thermal) interact via symmetric cross-attention within standard Transformer blocks (multi-head attention, feed-forward network, and LayerNorm). The block is used off-the-shelf in our pipeline and is not part of the proposed contributions.

As illustrated in Figure 3, the adopted CFT jointly processes RGB and thermal representations in a unified attention space. Let $F_{R}^{4},\;F_{T}^{4}\in R^{H\times W\times C}$ denote the RGB and thermal features at C4 after backbone extraction and MSFR pre-conditioning. Following a 1×1 projection for channel alignment, each feature map is flattened into a sequence of N=HW tokens and augmented with positional encoding and modality-specific embeddings, as defined in Equations (12) and (13).

(12) $$\begin{array}{cc} Z_{R}^{\left(0\right)} & =\mathrm{vec}\;\left(F_{R}^{4}\right)+P+E_{R} \end{array}$$

(13) $$\begin{array}{cc} Z_{T}^{\left(0\right)} & =\mathrm{vec}\;\left(F_{T}^{4}\right)+P+E_{T} \end{array}$$

where vec(·) reshapes the spatial feature maps into token sequences, *P* denotes a two-dimensional positional encoding broadcast along the sequence, and $E_{R}$ and $E_{T}$ are learnable modality embeddings for RGB and thermal tokens, respectively.

The CFT module comprises a stack of *L* bidirectional cross-attention layers. In each layer, RGB tokens attend to thermal keys and values, and thermal tokens attend symmetrically to RGB keys and values. Multi-head attention, residual connections, and layer normalization are applied following the standard Transformer formulation, with the attention operation given by Equation (14).

(14) $$\begin{array}{c} \mathrm{Attn}\left(Q,K,V\right)=\mathrm{softmax}\;\left(\frac{QK^{⊤}}{\sqrt{d}}\right)V \end{array}$$

where *Q*, *K*, and *V* denote the query, key, and value matrices derived from input features, and *d* is the scaling factor equal to the dimension per attention head.

This symmetric interaction scheme allows each modality to query and be guided by the other within the same attention space, thereby facilitating bidirectional information exchange between RGB and thermal representations. Compared with static or unidirectional fusion, such cross-modal interaction is better suited to capturing long-range semantic correspondence under illumination variation and partial occlusion.

After *L* layers of interaction, the output sequences $Z_{R}^{\left(L\right)}$ and $Z_{T}^{\left(L\right)}$ are reshaped back to spatial feature maps and fused along the channel dimension through a 1×1 projection, yielding the following cross-modal representation at C4:

(15) $$\begin{array}{c} F_{\times }^{4}=\phi_{1\times 1}\;\left([\;\mathrm{unvec}\left(Z_{R}^{\left(L\right)}\right)\;∥\;\mathrm{unvec}\left(Z_{T}^{\left(L\right)}\right)\;]\right) \end{array}$$

where unvec(·) restores the spatial layout, [·∥·] denotes channel-wise concatenation, and $F_{\times }^{4}$ is the fused C4 feature.

The fused representation preserves the spatial resolution at C4 and conforms to the channel interface expected by the subsequent stage. Nevertheless, attention-based interaction may still retain channel-wise redundancy or residual modality bias, particularly in cluttered or illumination-sensitive scenes. These responses are further handled by the AFR module at C5, which performs lightweight post-fusion recalibration before multi-scale aggregation.

From a computational perspective, the attention complexity scales as $O(N^{2})$ with N=HW. Deploying CFT at the C4 stage provides a practical trade-off between sequence length and semantic richness. Compared with earlier high-resolution stages, C4 reduces the token number and attention cost; compared with deeper low-resolution stages, it retains more spatial detail that is useful for small or distant pedestrians. Overall, in our framework, CFT functions as an adopted intermediate correspondence modeling component, while the main architectural novelty lies in the coordinated pre-fusion refinement and post-fusion recalibration surrounding it. Because CFT is adopted as an off-the-shelf interaction component rather than redesigned in this work, we keep its original attention-head setting and Transformer depth instead of conducting an additional hyperparameter search over these internal configurations. This choice helps avoid conflating the effect of CFT retuning with the contribution of the proposed MSFR and AFR modules. A more systematic sensitivity study of the number of attention heads and Transformer layers, especially with respect to small-target detection accuracy and computational cost, is left for future work.

### 3.4. Adaptive Feature Recalibration (AFR)

**Figure 4:**
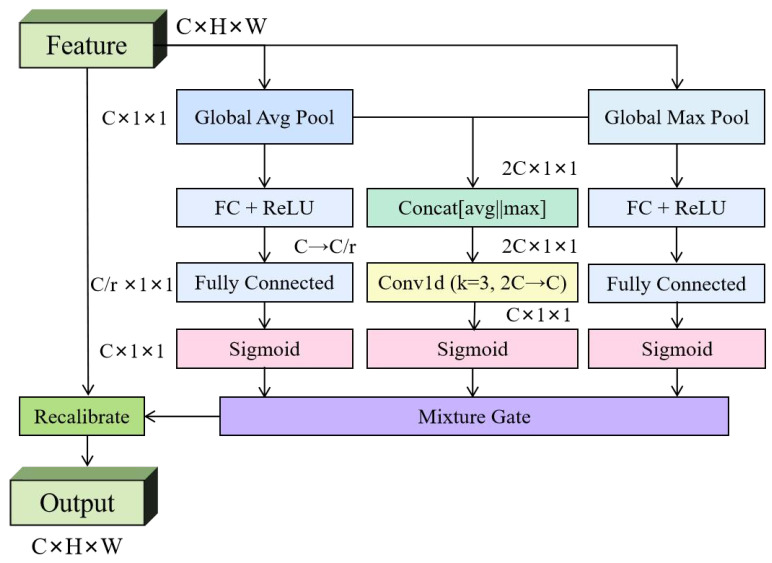
Illustration of the proposed AFR module. Given the fused feature *U*, global average pooling and global max pooling generate two channel descriptors. Each descriptor is processed by a two-layer gating network, while their concatenation is fed into a lightweight one-dimensional convolution to model local channel interactions. The three gating responses are then integrated to produce the final channel weights, which recalibrate the fused feature through a residual formulation.

After cross-modal interaction at stage C4, the fused representation already contains complementary information from RGB and thermal modalities. However, attention-based interaction may still retain background-dominated activations or modality-biased channel responses under illumination variation, sensor noise, and cluttered surroundings. If these responses are directly propagated to subsequent feature aggregation stages, weak or small-scale pedestrian cues may become less distinguishable. To alleviate this issue, we introduce an Adaptive Feature Recalibration (AFR) module at stage C5 to perform lightweight post-fusion channel recalibration before multi-scale aggregation, as illustrated in Figure 4. Let the AFR input be $U\in R^{H\times W\times C}$.

AFR is designed as a post-fusion recalibration module rather than an additional cross-modal fusion operator. The C5 stage is selected because the representation at this level has undergone cross-modal interaction and contains relatively strong semantic information, while still serving as an important input to the detection neck. Therefore, recalibrating channel responses at this stage provides an opportunity to suppress residual redundancy and rebalance fused features before multi-scale aggregation without modifying the earlier interaction mechanism.

AFR first summarizes channel-wise responses using two complementary global statistics, namely average pooling and max pooling, as defined in Equations (16) and (17).

(16) $$\begin{array}{cc} z_{c}^{\mathrm{avg}} & =\frac{1}{HW}\sum_{i=1}^{H}\sum_{j=1}^{W}U_{c}\left(i,j\right) \end{array}$$

(17) $$\begin{array}{cc} z_{c}^{\max} & =\max_{i,j}U_{c}\left(i,j\right) \end{array}$$

where $U_{c}\left(i,j\right)$ denotes the feature value at spatial position (i,j) in channel *c* of the input feature map $U\in R^{H\times W\times C}$. The resulting descriptors $z^{\mathrm{avg}}$ and $z^{\max}$ are both *C*-dimensional channel vectors.

The average descriptor reflects the overall activation level of each channel, whereas the max descriptor highlights sparse but salient responses. These two statistics provide complementary information for estimating channel importance: the former offers a global summary of fused responses, while the latter preserves localized peak activations that may be relevant to small targets or high-contrast thermal regions.

Based on these descriptors, AFR employs two parallel gating branches to compute channel-wise weights independently from average-based and max-based statistics, as defined in Equations (18) and (19).

(18) $$\begin{array}{cc} s^{\mathrm{avg}} & =\sigma \;\left(W_{2}^{\mathrm{avg}}\;\delta \left(W_{1}^{\mathrm{avg}}z^{\mathrm{avg}}\right)\right) \end{array}$$

(19) $$\begin{array}{cc} s^{\max} & =\sigma \;\left(W_{2}^{\max}\;\delta \left(W_{1}^{\max}z^{\max}\right)\right) \end{array}$$

where $z^{\mathrm{avg}}$ and $z^{\max}$ are the average-pooled and max-pooled channel descriptors from Equations (16) and (17), $W_{1}^{\left\{\cdot \right\}}$ and $W_{2}^{\left\{\cdot \right\}}$ are the learnable weights of the two linear layers in each branch, δ denotes the ReLU activation, and σ is the sigmoid function. In both gating branches, the channel descriptor is first compressed from *C* to C/r and then expanded back to *C*, where *r* denotes the shared channel reduction ratio.

While average- and max-based descriptors provide global statistics, they do not explicitly characterize local dependencies among neighboring channel responses. Since fused multispectral features may contain correlated activations across related channels, AFR introduces a third interaction branch operating along the channel dimension. Specifically, the two descriptors are concatenated and processed by a one-dimensional convolution, as formulated in Equations (20) and (21).

(20) $$\begin{array}{cc} \tilde{z} & =[\;z^{\mathrm{avg}}\;∥\;z^{\max}\;] \end{array}$$

(21) $$\begin{array}{cc} s^{\mathrm{conv}} & =\sigma \;\left(\mathrm{Conv}1D(\tilde{z})\right) \end{array}$$

where [·∥·] denotes channel-wise concatenation. The concatenated descriptor $\tilde{z}$ has dimension 2C, and Conv1D denotes a one-dimensional convolution with kernel size k=3 that maps the 2C-dimensional descriptor back to *C* channels for lightweight local channel interaction modeling. The sigmoid function σ is then used to generate the corresponding channel weight response.

This interaction branch complements the two global-statistics branches by introducing lightweight channel neighborhood modeling. In this way, AFR jointly considers average-level response strength, peak-level saliency, and local inter-channel dependency when estimating post-fusion recalibration weights.

The three gate vectors are then fused through a learnable mixture to produce the final channel weight vector, as defined in Equation (22).

(22) $$\begin{array}{c} s=\sigma \;\left(W_{f}[\;s^{\mathrm{avg}}\;∥\;s^{\max}\;∥\;s^{\mathrm{conv}}\;]\right) \end{array}$$

where $s^{\mathrm{avg}}$, $s^{\max}$, and $s^{\mathrm{conv}}$ are the three *C*-dimensional gate vectors from Equations (18)–(21), [·∥·∥·] denotes their channel-wise concatenation, and $W_{f}$ is a learnable fusion matrix that projects the concatenated 3C-dimensional descriptor to the final *C*-dimensional channel weight vector *s*.

Finally, channel-wise scaling combined with a residual connection yields the AFR output, as formulated in Equation (23).

(23) $$\begin{array}{c} Y_{\mathrm{AFR}}=U+s⊙U \end{array}$$

where *U* is the input feature map, *s* is the final channel weight vector from Equation (22), and ⊙ denotes element-wise multiplication along the channel dimension.

The residual formulation preserves the original fused representation while allowing adaptive channel-wise adjustment, and it keeps both spatial resolution and channel dimensionality unchanged. Compared with conventional channel attention mechanisms that mainly rely on a single pooling statistic or a fixed combination strategy, AFR is tailored to post-fusion multispectral features by integrating complementary global descriptors with lightweight local channel interaction. This design aims to reduce background-dominated or modality-skewed responses before multi-scale aggregation.

By construction, the AFR output $Y_{\mathrm{AFR}}$ (denoted as ${\hat{F}}^{5}$ in the architectural description) aligns directly with the neck interface for subsequent feature aggregation. Its additional computation mainly comes from two lightweight gating branches, a one-dimensional channel interaction branch, and a small fusion projection. The effectiveness and computational overhead of AFR are further examined in the experimental section. The three AFR branches are designed to provide complementary cues for post-fusion recalibration: average-based statistics summarize overall channel responses, max-based statistics preserve sparse salient activations, and the one-dimensional interaction branch models local channel dependencies. In this work, their contribution is examined at the module level through the overall architecture and ablation analysis. A more fine-grained interpretation of sample-wise branch responses or learned gating-weight distributions under different modality-imbalance conditions may provide additional insight and will be considered in future work.

### 3.5. Feature Aggregation and Training Objective

After multi-scale refinement at C3, cross-modal interaction at C4, and channel-wise recalibration at C5, the representative features are organized as complementary multi-level representations with compatible channel interfaces. Specifically, the MSFR-enhanced feature captures fine-grained structure and mid-level context, the CFT output encodes cross-modal semantic correspondence, and the AFR output rebalances channel importance after fusion. These complementary representations are unified before being forwarded to the detection neck.

Let $F_{\mathrm{MSFR}}$, $F_{\times }^{4}$, and ${\hat{F}}^{5}$ denote the outputs of MSFR, CFT, and AFR, respectively. These representative features are jointly forwarded to the neck network for multi-scale aggregation, as defined in Equations (24) and (25).

(24) $$\begin{array}{cc} F_{\mathrm{cat}} & =\left\{F_{\mathrm{MSFR}},\;F_{\times }^{4},\;{\hat{F}}^{5}\right\} \end{array}$$

(25) $$\begin{array}{cc} Y & =\phi_{\mathrm{head}}\;\left(\phi_{\mathrm{neck}}\left(F_{\mathrm{cat}}\right)\right) \end{array}$$

where $F_{\mathrm{cat}}$ denotes the collection of representative multi-level features, $\phi_{\mathrm{neck}}\left(\cdot \right)$ denotes the neck network for multi-scale aggregation, $\phi_{\mathrm{head}}\left(\cdot \right)$ denotes the detection head, and *Y* is the final detection output.

The neck performs top–down and bottom–up feature fusion to preserve spatial hierarchy, and the detection head predicts class probabilities and bounding boxes accordingly. This aggregation step unifies complementary information from the detail refinement domain (MSFR), the cross-modal alignment domain (CFT), and the importance recalibration domain (AFR) into a shared feature representation for downstream detection under weak-texture, low-contrast, and long-range scenarios.

The overall framework is trained using the standard detection loss of the adopted single-stage detector, which combines classification, objectness, and bounding box regression terms. The training objective is defined in Equation (26).

(26) $$L_{\det}=\alpha \;L_{\mathrm{cls}}+\beta \;L_{\mathrm{obj}}+\gamma \;L_{\mathrm{box}}$$

where $L_{\mathrm{cls}}$, $L_{\mathrm{obj}}$, and $L_{\mathrm{box}}$ denote the classification, objectness, and bounding box regression losses, respectively, and α, β, and γ are their corresponding weights. The concrete formulations of these loss terms are kept consistent with the adopted detection head.

By using the standard detection objective, the proposed framework isolates the architectural design itself, namely multi-scale refinement before fusion, explicit cross-modal interaction, and adaptive channel recalibration after fusion. This setting helps attribute the observed performance differences primarily to feature representation and fusion quality rather than to additional optimization constraints or auxiliary supervision.

## 4. Experiments

### 4.1. Datasets and Evaluation Metrics

We evaluate the proposed method on three widely used RGB–thermal benchmarks, namely KAIST [17], FLIR-aligned [19], and LLVIP [18]. Among them, KAIST and LLVIP are pedestrian-focused datasets, while FLIR-aligned is used as a road-scene RGB–thermal object detection benchmark covering person, car, and bicycle. These datasets span different illumination conditions, scene layouts, and target scales, and thus provide a practical basis for evaluating the proposed method across both pedestrian-focused and broader road-scene multispectral detection settings. Unless otherwise stated, all experiments in this work are conducted on fixed hold-out splits rather than k-fold cross-validation.

**Figure 5:**
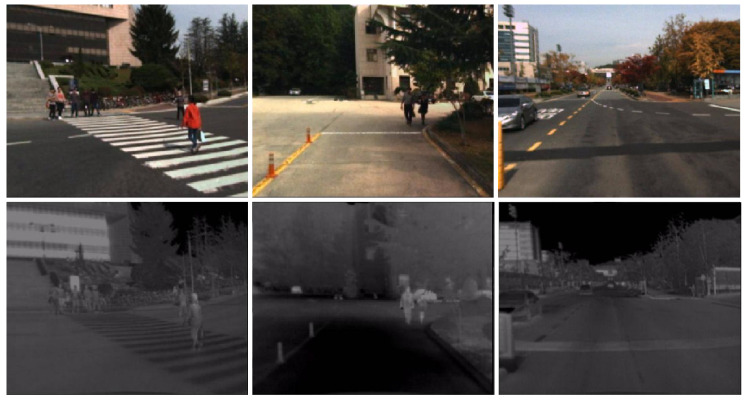
Examples from the KAIST dataset. The top row shows RGB images and the bottom row shows the corresponding thermal images from different traffic scenes.

The KAIST dataset [17] is a widely used benchmark for RGB–thermal pedestrian detection. It contains aligned visible–thermal image pairs captured in daytime and nighttime street scenes using a beam-splitter-based imaging system that provides accurate spatial correspondence between the two modalities. Following common practice in multispectral pedestrian detection, we adopt the cleaned annotation setting and use the Reasonable configuration as the primary evaluation protocol for KAIST. Example image pairs are shown in Figure 5.

For the FLIR benchmark [19], we use the FLIR-aligned version in this work. Compared with the original FLIR ADAS release, this version retains only aligned visible–thermal image pairs and has been adopted in a number of recent multispectral detection studies. In our experiments, the FLIR-aligned dataset contains 5142 image pairs, of which 4129 are used for training and 1013 are used for testing. Following the commonly adopted aligned-FLIR protocol used by related RGB–thermal object detection studies, three object categories are evaluated: person, car, and bicycle. Unless otherwise stated, all FLIR results reported in this paper refer to this aligned version and are presented as overall mAP across these three categories.

The LLVIP dataset [18] focuses on low-light pedestrian detection and provides strictly aligned RGB–thermal image pairs captured by stereo cameras. Owing to its accurate temporal and spatial correspondence, LLVIP is suitable for evaluating cross-modal complementarity under low-illumination conditions. In this work, we follow the commonly adopted pedestrian-detection split and use 12,025 image pairs for training and 3463 image pairs for testing.

Since the three datasets follow different benchmark conventions, we report dataset-specific evaluation metrics accordingly. For the KAIST dataset, we report the log-average Miss Rate (MR, %) computed over the false-positives-per-image (FPPI) range $\left[10^{-2},10^{0}\right]$, where lower values indicate better detection performance. For the FLIR-aligned dataset, we report mean Average Precision at the IoU threshold 0.5 (mAP@50) and COCO-style mean Average Precision averaged over IoU thresholds from 0.5 to 0.95 (mAP@0.5:0.95), computed over the three evaluated categories: person, car, and bicycle. For the LLVIP dataset, which focuses exclusively on pedestrians, the same mAP metrics are reported for pedestrian detection.

### 4.2. Implementation Details

All experiments were conducted on Ubuntu 20.04 using Python 3.8, PyTorch 2.0.0, and CUDA 11.8 on a single NVIDIA GeForce RTX 4090 GPU with 24 GB memory (NVIDIA Corporation, Santa Clara, CA, USA). Unless otherwise specified, all in-house models were trained and evaluated at an input resolution of 640×640 under a single-scale setting. Training was performed for 50 epochs using SGD with an initial learning rate of 0.01, a momentum of 0.937, a weight decay of $5\times 10^{-4}$, and a batch size of 8. No test-time augmentation was applied during inference.

For controlled evaluation within our implementation pipeline, the baseline denotes a lightweight dual-stream RGB–thermal detector that uses the same backbone, neck, detection head, input resolution, and training protocol as the proposed model, but does not include the MSFR, CFT, or AFR modules. This setting provides a consistent reference for examining the performance changes associated with the proposed refinement–interaction–recalibration design. The proposed model is constructed by introducing MSFR for pre-interaction feature refinement, the adopted CFT for cross-modal correspondence modeling, and AFR for post-interaction feature recalibration into this baseline architecture.

In addition, an in-house YOLOv5 (RGB) model is included only as a contextual single-modality reference for comparison with the RGB–thermal detection settings. It is not used as a baseline for assessing the proposed fusion pipeline. The baseline, the proposed model, the in-house YOLOv5 (RGB) reference, and all ablation variants were trained under the same optimization settings whenever they were evaluated within our implementation pipeline.

Inference throughput (FPS) was measured on the same hardware and codebase with batch size fixed to 1, and is reported only for our in-house models. Unless otherwise stated, the implementation-dependent results reported in this work are obtained from our in-house evaluation setting, while the remaining comparison results in the following tables are taken from the corresponding publications and are provided as benchmark-level contextual references.

### 4.3. Comparison Experiment

#### 4.3.1. Experiments on the KAIST Dataset

To evaluate the proposed method on multispectral pedestrian detection, we conduct experiments on the KAIST benchmark under the Reasonable setting, and the comparison results are summarized in Table 1. As shown in the table, the proposed method achieves an MR of 11.18%, reducing the miss rate of the in-house lightweight baseline from 16.95% to 11.18%, which suggests that the proposed refinement–interaction–recalibration design is beneficial for improving the baseline detector under our implementation setting. Compared with earlier conventional methods such as ACF and Halfway Fusion, our method also obtains lower miss rates, indicating the potential value of learned RGB–thermal feature modeling for challenging pedestrian scenes. Meanwhile, several published detectors, including MBNet, MLPD, CFT, and ICAFusion, report lower MR values than ours; therefore, the KAIST results are mainly used to support the effectiveness of the proposed design in improving the accuracy–efficiency trade-off of a compact multispectral pedestrian detector, rather than to claim absolute state-of-the-art performance.

#### 4.3.2. Experiments on the FLIR Dataset

To further evaluate the proposed method in RGB–thermal road-scene detection, we conduct experiments on the FLIR-aligned benchmark under the commonly used three-category evaluation setting covering person, car, and bicycle, and the comparison results are summarized in Table 2. Under our implementation setting, the proposed method achieves 84.2% mAP@50 and 45.9% mAP@0.5:0.95, improving the in-house baseline by 4.2 and 4.9 points, respectively, which suggests that the proposed stage-wise fusion design may enhance multispectral feature representation and localization quality relative to the baseline configuration. Relative to the selected published RGB–thermal detectors listed in Table 2, our method shows competitive benchmark-level performance and remains numerically close to GM-DETR; however, these cross-paper values are quoted from prior publications and are mainly used for contextual comparison rather than as strictly controlled head-to-head evidence. In addition, the proposed model obtains higher values than the in-house single-modality YOLOv5 (RGB) reference on both reported metrics, which is consistent with the expected benefit of exploiting complementary visible and thermal information, while its compact size of 2.94 M parameters further provides supplementary support for a favorable balance between detection quality and model compactness in road-scene multispectral detection.

#### 4.3.3. Experiments on the LLVIP Dataset

To further evaluate the proposed method under low-illumination pedestrian detection conditions, experiments are conducted on the LLVIP benchmark, and the comparison results are summarized in Table 3. Under our implementation setting, the proposed method achieves 98.5% mAP@50 and 70.5% mAP@0.5:0.95, improving the in-house baseline by 0.6 and 1.3 points, respectively. Although the baseline already provides strong performance on LLVIP, the proposed method still yields consistent gains under the same training and evaluation setting. Relative to the selected published multispectral detectors listed in Table 3, our method shows competitive benchmark-level results, particularly on the stricter mAP@0.5:0.95 metric, while these literature-reported values are mainly used to position the proposed method within the broader benchmark landscape. Moreover, the proposed RGB–thermal detector performs better than the in-house single-modality YOLOv5 (RGB) reference on both metrics, supporting the usefulness of visible–thermal complementarity for low-light pedestrian detection and suggesting that the proposed refinement–interaction–recalibration design is associated with consistent improvements in challenging pedestrian-centric scenes.

### 4.4. Ablation Study

We conduct a module-level ablation study on the KAIST dataset under the same training and evaluation setting to examine the contribution of each component in the proposed pipeline. Starting from the lightweight baseline detector, we evaluate the effects of the adopted Cross-Modality Fusion Transformer (CFT), the proposed Multi-scale Feature Refinement (MSFR) module, and the proposed Adaptive Feature Recalibration (AFR) module. In addition to the single-module settings, all pairwise combinations and the complete configuration are also considered. The results are summarized in Table 4.

As shown in Table 4, each individual component improves the baseline to a certain extent. Adding CFT reduces MR from 16.95% to 14.98%, while introducing MSFR or AFR alone lowers MR to 14.30% and 14.00%, respectively. These results suggest that cross-modal interaction, pre-interaction feature refinement, and post-interaction recalibration each provide useful contributions under the same implementation setting. The corresponding parameter increases remain moderate, indicating that these gains are obtained without substantially enlarging the lightweight detector.

The pairwise configurations further show complementary behavior among the three components. Combining CFT with MSFR yields an MR of 13.20%, which is consistent with the design motivation that refining modality-specific features before interaction can benefit subsequent cross-modal modeling. Combining CFT with AFR reduces MR to 12.80%, suggesting that recalibration after interaction is also helpful for improving fused representations. The MSFR + AFR setting achieves an MR of 12.30%, which is the best result among the pairwise configurations and indicates that pre-refinement and post-recalibration can provide complementary improvements even without explicit Transformer-based interaction.

The complete configuration with CFT, MSFR, and AFR achieves the lowest MR of 11.18%. Relative to the baseline, this corresponds to a reduction of approximately 34%, while the model size remains 2.94 M parameters and the inference speed is 151.2 FPS. Although the added modules introduce a measurable computational cost compared with the baseline, the full model still maintains its real-time inference capability. Overall, the ablation results provide evidence that the refinement–interaction–recalibration pipeline is beneficial for the lightweight detector and that the three components contribute in a complementary manner.

### 4.5. Statistical Stability Analysis

To provide a supplementary assessment of result stability, we repeated the training of the in-house baseline and our model using three different random seeds (42, 1024, and 2026), while keeping the remaining settings unchanged. Following the main evaluation protocol of each benchmark, we report MR on KAIST and mAP@50 on FLIR-aligned and LLVIP. These metrics are used here to provide a concise view of repeated-run behavior while avoiding additional variability associated with stricter IoU-based measures. The corresponding mean and standard deviation are summarized in Table 5.

As shown in Table 5, the proposed method achieves better mean performance than the baseline across the three benchmarks within this supplementary three-seed evaluation, and the observed standard deviations remain relatively small. Specifically, on KAIST, MR decreases from 16.98±0.12 to 11.21±0.08; on FLIR-aligned, mAP@50 increases from 79.98±0.18 to 84.17±0.11; and on LLVIP, it increases from 97.88±0.05 to 98.47±0.04. This analysis is intended as a supplementary stability check rather than a formal statistical significance test. Nevertheless, the results provide additional support that the improvements reported in the main experiments are reproducible across several random initializations under our implementation setting.

### 4.6. Misalignment Sensitivity Analysis

To further examine the influence of cross-modal registration errors, we conduct an additional sensitivity analysis on the FLIR-aligned benchmark, and the results are reported in Table 6. In this analysis, artificial spatial offsets of 0, 1, 2, 4, and 8 pixels are introduced between the paired RGB and thermal inputs to simulate increasing levels of cross-modal misalignment, while the remaining evaluation setting is kept unchanged. As the shift magnitude increases, the performance of both the baseline and our model gradually declines, indicating that accurate cross-modal correspondence remains important for RGB–thermal detection. Compared with the baseline, the proposed method retains higher scores across the tested offsets, and the degradation under small spatial shifts is relatively moderate. These results suggest that the proposed refinement–interaction–recalibration design may provide a certain degree of tolerance to mild artificial misalignment, although larger offsets still lead to noticeable performance degradation. Therefore, the current results should be interpreted as a supplementary sensitivity analysis rather than as a complete solution to real-world cross-modal registration errors.

### 4.7. Qualitative Visualization

This section presents the qualitative results of representative RGB–thermal scenes to provide complementary visual observations for the quantitative evaluation. The selected examples cover several challenging yet typical cases, including long-range pedestrians in daytime scenes, pedestrians with oblique or side-facing poses, and pedestrians under low-illumination or nighttime conditions. These scenarios are informative for qualitative analysis because the relative saliency of RGB and thermal cues may vary substantially and background interference is often more pronounced.

**Figure 6:**
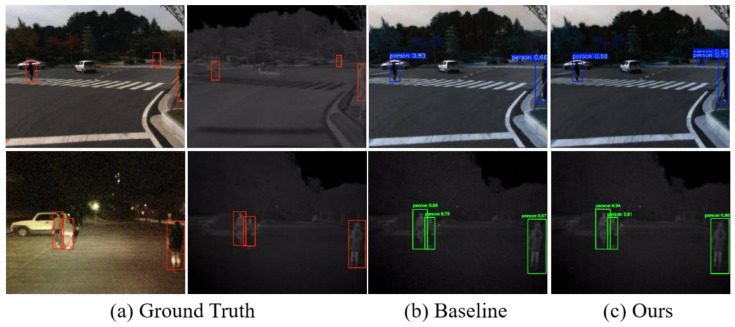
Detection results on the KAIST dataset. (**a**) shows the annotated RGB–thermal image pairs, (**b**) shows the detection results of the baseline model, and (**c**) shows the results of the proposed method. Red bounding boxes in (**a**) denote ground-truth annotations, while colored bounding boxes in (**b**,**c**) denote predicted detections. In the first row of (**c**), the additional predicted bounding box marks a target that is missed by the baseline but detected by the proposed method.

Figure 6 compares the detection results of the baseline model and the proposed method on the same scenes. Each row corresponds to one representative example, while the three column groups show (a) the aligned RGB–thermal image pairs with ground-truth annotations, (b) the detections produced by the baseline model, and (c) the detections produced by the proposed method. In these examples, the baseline model exhibits missed detections or less precise localization in several difficult cases, especially for small or distant pedestrians, diagonally walking pedestrians, and nighttime scenes with weak illumination. By comparison, the proposed method recovers some targets that are missed or loosely localized by the baseline and shows fewer visually obvious responses to distracting background structures such as road markings and headlights. These observations are consistent with the quantitative improvements reported in the comparison and ablation experiments.

**Figure 7:**
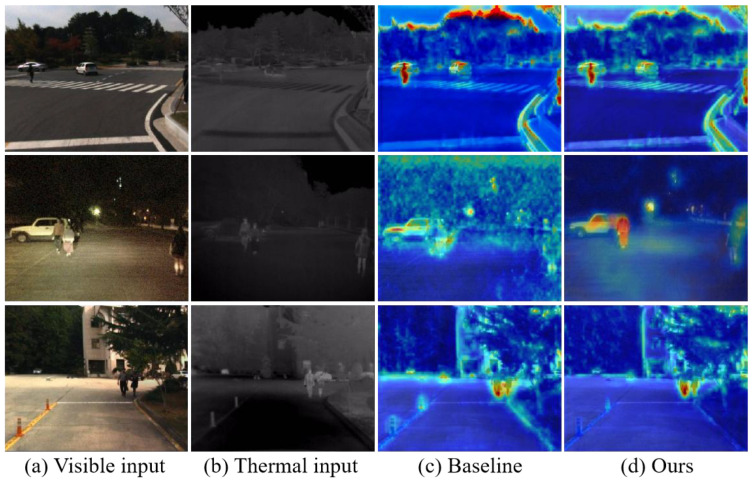
Visualization of feature responses on representative multispectral scenes. (**a**,**b**) are the visible and thermal inputs, respectively. (**c**) shows the activation map of the baseline model, while (**d**) presents the activation map of the proposed method. In these examples, the proposed model exhibits more concentrated responses around pedestrian regions and less dispersed activation over some background areas.

Figure 7 further visualizes the feature-response maps of the baseline model and the proposed method. In each row, subfigures (a) and (b) show the RGB and thermal inputs, respectively, illustrating that pedestrian saliency may differ across modalities under varying illumination conditions. Subfigure (c) presents the activation map of the baseline model, which shows relatively dispersed responses over some background regions, such as sky areas, vegetation, and bright road surfaces. Subfigure (d) shows the activation map of the proposed method, where the responses in these examples appear more concentrated around pedestrian regions and less scattered across irrelevant background areas. Although these visualizations are qualitative and do not isolate the effect of each individual module, they provide additional evidence that the complete refinement–interaction–recalibration pipeline tends to produce more task-focused feature responses than the baseline in representative challenging scenes.

Taken together, the detection examples and activation maps offer qualitative observations that align with the numerical results. The proposed method shows more favorable visual behavior in several difficult pedestrian scenarios, particularly for small targets, long-range pedestrians, and low-light cases. These results should be interpreted as complementary visual support rather than as a standalone module-wise analysis, but they nevertheless help illustrate how the full model differs from the baseline in representative multispectral detection settings.

## 5. Conclusions

In this work, we investigated RGB–thermal multispectral pedestrian detection under challenging conditions such as low illumination, long-range observation, and cluttered backgrounds. Starting from a lightweight dual-stream RGB–thermal baseline, we developed a stage-wise fusion pipeline that combines a proposed Multi-Scale Feature Refinement (MSFR) module, an adopted Cross-Modality Fusion Transformer (CFT), and a proposed Adaptive Feature Recalibration (AFR) module. Rather than redesigning the Transformer-based interaction operator, the proposed method focuses on improving the fusion process surrounding cross-modal interaction: MSFR refines modality-specific representations before interaction, CFT models semantic correspondence between RGB and thermal features, and AFR recalibrates fused responses after interaction. This refinement–interaction–recalibration design aims to improve lightweight multispectral fusion while preserving a compact model size and real-time inference capability.

Experiments on three public RGB–thermal benchmarks, including the pedestrian-focused KAIST and LLVIP datasets together with the FLIR-aligned road-scene benchmark, show that the proposed method improves the in-house lightweight baseline across the reported evaluation settings. The benchmark results and qualitative examples further suggest favorable behavior in low-light, long-range, and small-target pedestrian scenarios. The ablation results indicate that MSFR, CFT, and AFR provide complementary contributions within the overall pipeline, while the visualization results show that the proposed model tends to generate more concentrated responses around pedestrian regions with reduced activation on irrelevant background structures.

Several limitations remain. First, comparisons with published methods are mainly benchmark-level references rather than strictly controlled head-to-head reimplementations under a unified pipeline. Second, the current statistical stability analysis is based on a three-seed supplementary check and should not be interpreted as a formal significance test. Third, the framework still assumes reasonably aligned RGB–thermal inputs; although the sensitivity analysis suggests improved tolerance to mild artificial shifts, it does not fully cover more complex real-world misalignment or registration errors. Fourth, because CFT is adopted without internal redesign and AFR is evaluated mainly at the module level, finer-grained analyses of CFT hyperparameter sensitivity and AFR branch-wise gating behavior remain to be explored. In addition, robustness under more extreme illumination changes, severe thermal sensor noise, and cross-dataset generalization settings warrants further investigation. Future work may therefore focus on more rigorous cross-method reproduction, broader statistical verification, alignment-robust fusion, deeper interpretability analysis, and improved generalization to more challenging multispectral scenarios.

## Figures and Tables

**Table 1:** Comparison on the KAIST dataset.

Method	Modality	MR (%) ↓
ACF [36]	R + T	27.6
Halfway Fusion [20]	R + T	25.2
MBNet [37]	R + T	8.13
MLPD [38]	R + T	7.61
CFT [22]	R + T	7.45
ICAFusion [39]	R + T	7.17
Baseline ^†^	R + T	16.95
**Ours** ^†^	**R + T**	**11.18**

*Note:* ^†^ denotes in-house results obtained under our implementation setting. Results of the other methods are reported from the corresponding publications and are included for contextual benchmark reference. The downward arrow indicates that lower values are better.

**Table 2:** Comparison on the FLIR-aligned dataset.

Method	Modality	mAP@50 (%)	mAP@0.5:0.95 (%)
YOLOv5 [40] (RGB) ^†^	RGB	68.4	32.2
YOLO-MS [41]	R + T	75.2	38.3
MRD-YOLO [42]	R + T	76.5	40.9
CFT [22]	R + T	78.3	40.2
ICAFusion [39]	R + T	79.2	41.4
Baseline ^†^	R + T	80.0	41.0
GM-DETR [43]	R + T	83.9	45.8
**Ours** ^†^	**R + T**	**84.2**	**45.9**

*Note:* ^†^ denotes in-house results obtained under our implementation setting. Results of the other methods are reported from the corresponding publications and are included for contextual benchmark reference. The FLIR-aligned results are reported as overall mAP over three evaluated categories: person, car, and bicycle. YOLOv5 (RGB) is included only as a single-modality reference rather than as the baseline of the proposed fusion pipeline.

**Table 3:** Comparison on the LLVIP dataset.

Method	Modality	mAP@50 (%)	mAP@0.5:0.95 (%)
YOLOv5 [40] (RGB) ^†^	RGB	89.8	48.8
CFT [22]	R + T	97.5	63.6
CSAA [44]	R + T	94.3	59.2
ICAFusion [39]	R + T	98.4	64.5
GM-DETR [43]	R+T	97.4	70.2
DAMSDet [14]	R + T	97.9	69.6
Fusion-Mamba [5]	R + T	97.0	64.3
Baseline ^†^	R + T	97.9	69.2
**Ours** ^†^	**R + T**	**98.5**	**70.5**

*Note:* ^†^ denotes in-house results obtained under our implementation setting. Results of the other methods are reported from the corresponding publications and are included for contextual benchmark reference. YOLOv5 (RGB) is included only as a single-modality reference rather than as a baseline of the proposed fusion pipeline.

**Table 4:** Module-level ablation study on the KAIST dataset under the Reasonable evaluation setting. The metric is the log-average Miss Rate (MR, %) computed over FPPI $\in [10^{-2},10^{0}]$, where lower values indicate better performance.

Group	CFT	MSFR	AFR	MR (%) ↓	Params (M)	FPS
1				16.95	2.37	233.4
2	✓			14.98	2.66	186.7
3		✓		14.30	2.68	205.4
4			✓	14.00	2.63	214.7
5	✓	✓		13.20	2.87	164.3
6	✓		✓	12.80	2.85	178.1
7		✓	✓	12.30	2.83	189.0
8	✓	✓	✓	**11.18**	2.94	151.2

*Note:* ✓ indicates that the corresponding module is used. The downward arrow indicates that lower MR values are better.

**Table 5:** Statistical stability of the baseline and our model under three random seeds.

Dataset	Metric	Baseline	Ours
KAIST	MR (%) ↓	16.98 ± 0.12	**11.21 ± 0.08**
FLIR-aligned	mAP@50 (%) ↑	79.98 ± 0.18	**84.17 ± 0.11**
LLVIP	mAP@50 (%) ↑	97.88 ± 0.05	**98.47 ± 0.04**

*Note:* The downward arrow indicates that lower values are better, while the upward arrow indicates that higher values are better.

**Table 6:** Sensitivity analysis of baseline and our model to artificial cross-modal misalignment on FLIR-aligned dataset.

Shift	Baseline		Ours	
**(Pixels)**	**mAP@50**	**mAP@0.5:0.95**	**mAP@50**	**mAP@0.5:0.95**
0	80.0	41.0	84.2	45.9
1	79.4	40.5	83.6	45.3
2	78.6	39.6	82.8	44.4
4	76.3	37.8	80.6	42.4
8	72.8	34.8	77.1	39.2

## Data Availability

The data presented in this study are available from the authors upon reasonable request. Considering the current project-related arrangements and the need to further organize third-party dependencies and project-specific implementation files, relevant code materials related to the proposed MSFR and AFR modules may be made available from the corresponding author upon reasonable request. A broader public release may be considered when the project conditions and code organization are appropriate.
